# STAT3 phosphorylation at Tyr705 affects DRP1 (dynamin-related protein 1) controlled-mitochondrial fission during the development of apoptotic-resistance in pulmonary arterial endothelial cells

**DOI:** 10.1007/s13258-024-01522-w

**Published:** 2024-05-11

**Authors:** Han Zhang, Li Chen, Jiachen Li, Jiashu Sun, Qixu Zhao, Sheng Wang, Gang Li

**Affiliations:** Beijing Anzhen Hospital, Capital Medical University, 2 Anzhen Road, Beijing, 100029 China

**Keywords:** Pulmonary arterial hypertension 1, Mitochondrial fission 2, Apoptosis-resistance 3, Endothelial cell 4, STAT3 5

## Abstract

**Background:**

The apoptosis-resistant pulmonary arterial endothelial cells (PAECs) are known to be major players in the pulmonary remodeling of pulmonary arterial hypertension (PAH) and exhibit an abnormal metabolic profile with mitochondrial dysfunction. Mitochondrial fission has been shown to regulate the apoptosis of several cell types, but this is largely unexplored in the PAECs.

**Objective:**

The roles of mitochondrial fission control by Dynamin related protein-1 (DRP1) in the development of PAECs apoptosis suppression were investigated in present study and the potential mechanisms behind this were furtherly explored.

**Methods:**

The mitochondrial morphology was investigated in PAECs from PAH rats with the pulmonary plexiform lesions, and the relations of it with DRP1 expression and apoptosis were furtherly identified in apoptosis-resistant PAECs induced by hypoxia. PAECs were isolated from rats with severe PAH and from normal subjects, the apoptotic-resistant PAECs were induced by hypoxia. DRP1 gene knockdown was achieved via DRP1-siRNA, DRP1 and STAT3 phosphorylation were blocked using its inhibitors, respectively. Apoptosis was analyzed by flow cytometry, and mitochondrial morphology was investigated by transmission electron microscope and confocal microscopy.

**Results:**

The PAECs isolated from PAH rats with the pulmonary plexiform-like lesions and displayed lower apoptotic rate with increased DRP1 expression and mitochondrial fragmentation. In addition, similar observations were achieved in apoptosis-resistant PAECs induced by hypoxia. Targeting DRP1 using siRNA and pharmacologic blockade prevented the mitochondrial fission and subsequent apoptotic resistance in PAECs under hypoxia. Mechanistically, STAT3 phosphorylation at Tyr705 was shown to be activated in both PAH and hypoxia-treated PAECs, leading to the regulation of DRP1 expression. Of importance, targeting STAT3Tyr705 phosphorylation prevented DRP1 disruption on apoptosis in PAECs under hypoxia.

**Conclusions:**

These data indicated that STAT3 phosphorylation at Tyr705 impacted DRP1-controlled mitochondrial fission during the development of apoptosis-resistance in PAECs, suggesting mitochondrial dynamics may represent a therapeutic target for PAH.

**Supplementary Information:**

The online version contains supplementary material available at 10.1007/s13258-024-01522-w.

## Introduction

Pulmonary arterial hypertension (PAH) is an obstructive, arterial vasculopathy with dysfunctional pulmonary artery microvascular endothelial cells (PAECs), pulmonary artery smooth muscle cells, fibroblasts, and inflammatory cells, causing to the abnormal vasoconstriction and vascular remodeling and ultimately leading to fatal right ventricular failure (Archer et al. 2010). Among these abnormal cells, PAECs are central to the pulmonary vascular remodeling that increases pulmonary vascular resistance. Initial widespread PAECs apoptosis caused by the cascade of events culminated in the selection of apoptosis-resistant proliferating PAECs induces the release of mediators that cause vascular smooth muscle cells proliferation, resulting in complex precapillary pulmonary arteriolar lesions including neointima formation, plexiform and dilation lesions (Sakao et al. 2005; Xu and Erzurum 2011), which are the histopathologic hallmarks of irreversible PAH. The mechanisms involved in the development of apoptosis-resistant PAECs from abnormal apoptotic PAECs were largely unknown yet.

The hyperproliferative and apoptosis-resistant PAECs exhibit a metabolic profile strikingly like that of cancer cells, which show reliance upon glycolysis and shift away from oxidative phosphorylation for cellular energy production that named as the Warburg effect (Yu and Chan 2017). The apoptosis resistant PAECs from PAH showed hyperpolarized mitochondrial membrane potential (Δψm), observed from the Warburg effect as well (Pak 2013). It has been shown that intrinsic deficiencies in mitochondrial function in the metabolic shift to glycolysis in PAH and secondary abnormalities in mitochondrial function following endothelial dysfunction (Sharma et al. 2008). PAECs from patients with idiopathic pulmonary hypertension showed decreased mitochondrial dehydrogenase activity, mitochondrial number, mitochondrial DNA content and higher glycolytic rate compared to normal cells (Xu et al. 2007). It is increasingly realizing that dysregulated endothelial mitochondrial function drives primary aspects of PAH from inception to the end-stage, but the molecular details of this activity are not fully understood.

Mitochondria exist as a dynamic network comprising individual organelles that continuously join (fusion) and fragment (fission), a phenomenon known as mitochondrial dynamics. Fusion is mediated by the GTPases mitofusin-1 (MFN1), mitofusin-2 (MFN2), and optic atrophy-1, while fission is regulated by fission-1 and dynamin-related protein-1 (Drp1). The balance between them is very important for mitochondrial participation in crucial cellular pathophysiological processes. For example, fission plays key roles in mitosis, apoptosis, and autophagy, while fusion is a necessary adaptation to nutrient deficiency and increase of metabolic demand allowing transmission of transmembrane potential along interconnected mitochondria.

Several lines of evidence have shown that mitochondria dynamics were involved in the pulmonary artery remodeling via targeting pulmonary smooth muscle cells. Marsboom Get al. (2012) showed that hypoxia-inducible factor-1α activation in pulmonary smooth muscle cells from human PAH leads to mitochondrial fission by phosphorylation of Drp1 at serine 616, and hypoxia-inducible factor-1α inhibition reduced Drp1 activation, prevented fission, and reduced pulmonary smooth muscle cells proliferation. Both the Drp1 inhibitor Mdivi-1 and siDrp1 prevent mitotic fission and arrest PAH pulmonary artery muscle smooth cells at the G2/M interphase (Marsboom et al. 2012). Parra et al. (2017) reported that mitochondrial fusion triggered by the fatty acid oxidation inhibitor TMZ, the pharmacological Drp1 inhibitor Mdivi-1, or transduction with a mutant Drp1 all prevent mitochondrial dysfunction induced by hypoxia in human pulmonary smooth muscle cells. However, the reports of mitochondrial dynamics affecting PAECs in pulmonary vascular remodeling are rare.

In this study, we hypothesize that Drp1-mediated mitochondrial fission involved in the development of apoptosis-resistant PAECs from early apoptotic PAECs. The role of STAT3, a regulator of angiogenesis and cell survival, was investigated during this process to further explore the mechanism of Drp1-mediated mitochondrial fission, since it helps to promote an apoptosis-resistant environment through activation of survivin, NAFT, and Bcl-2, in addition to its directly regulation of mitochondrial function (Paulin et al. 2012).

## Materials and methods

### Animal model

SPF grade male Sprague–Dawley rats, 200-220 g in weight, were obtained from Beijing Weitong Lihua Laboratory Animal Technology Ltd. Co. (Beijing, China). The PAH rat model was performed by monocrotaline treatment (40 mg/kg, diluted in DMSO, i.p.) following left pneumonectomy as previously described (White et al. 2007). The control rats were received vehicle injection following sham procedure. The treated rats were housed in laboratory conditions with free access to food and tap water for three weeks. Hemodynamics measurements and cardiopulmonary pathology were used for confirmation of pulmonary arterial hypertension and plexiform-like lesions in pulmonary arteries.

### Cell culture and treatment

PAECs were isolated from PAH rats showing plexiform lesions in pulmonary arterioles and control rats. Pulmonary arteries were dissected to the distal small arterioles, and then the PAECs were isolated from pulmonary arterial tree as previously described by Chen et al. (1995). Cells were passaged at 70–80% confluence by dissociation from plates with 0.25% trypsin–EDTA (Invitrogen, Carlsbad, CA). The PAECs were identified by inverted microscope for observation of the morphology and growth behavior of characteristics, and flow cytometry using specific labeled monoclonal antibody CD31 and CD309. All experiments were performed on cells between passages 3 and 8.

Based on the literature, the Drp1 inhibitor Mdivi-1 (Xie et al. 2015) (25 μM, Enzo Life Sciences) and inhibitor of STAT3 phosphorylation AG490 (Zhou et al. 2019) (1 μM, Sigma-Aldrich, St. Louis, USA) were used for prevention studies, respectively. DMSO and Saline were used as vehicle control for Mdivi-1 and AG490, respectively.

### Small interfering RNA silenced Drp1 expression

To reduce the expression of Drp1 protein, PAECs were transfected with the small interfering RNA (siRNA), which were purchased from Santa Cruz Biotechnology, Inc. (Santa Cruz, USA). When the PAECs were cultured approximately 80% confluence, 1.5 μg Drp1-siRNA and 7.5 μl lipofectamine siRNA transfection reagent that diluted in serum-free medium were added. After 4 h, normal culture medium was added to the mixture and Drp1 protein expression was investigated after 48 h using western blot analysis. Nontargeted control siRNA (NTRNA) was used as the negative control for each experiment.

### Apoptosis-resistant induction and apoptosis assay

Apoptosis-resistant rat pulmonary vascular endothelial cells (AR group) were induced by the low oxygen (5% O_2_) cultivation for 24 h, as previously described (Cao et al. 2016). Apoptosis was quantified using an PE Annexin V/7-AAD apoptosis detection kit (BD, Franklin Lakes, NJ, USA) and Apo-ONE Homogeneous Caspase-3/7 Assay (Promega, Madison, Wisconsin, USA) in accordance with the manufacturer’s protocol.

### Mitochondrial morphology analysis

To observe the morphologic changes of the mitochondria, live PAECs mitochondria were labeled with 100 mol/L Mitotracker Green FM (Invitrogen, USA) for 30 min. Confocal images were collected with a Carl Zeiss LSM-5 Pascal 5 Axiovert 200 microscope, using a Plan-Apochromat 63 × /1.4 Oil DIC objective, and the LSM 5 3.3 software. In addition, PAECs were fixed, stained, dehydration, and then visualized with a Tecnai G2 SpiritBT electron microscope operated at 60 kV. Mitochondrial length was measured using the Image J software (NIH, Bethesda, MD), and mitochondrial number was counted and averaged in 30 cells/ per sample. Mitochondrial length of less than 1 μm was defined as fragmented.

### Western blotting analysis

Cell lines were processed for western blotting as previously described (Xie et al. 2015). Blotting was performed on 25 μg of protein from PAECs. Primary antibodies included Drp1 (Santa Cruz, USA), STAT3(Santa Cruz, USA), STAT3^Tyr705^(Santa Cruz, USA) and were diluted at 1:1,000. β-actin (Cell Signaling Technology, USA) was used as an internal control. The band intensity was scanned and quantified using Gel-Pro software (Media Cybernetics, USA).

### Immunofluorescence analysis

PAECs were fixed in 4% paraformaldehyde and immunolabeled using the antibodies. PAECs were fixed in 4% paraformaldehyde, permeabilized, blocked and incubated with a primary antibody Drp1 overnight at 4℃ and then a secondary antibody for 1 h. Images were obtained using a Leica digital microscope (DMI4000B).

### Flow cytometry analysis

Confluent cells were trypsinized, washed, and stained with PE Annexin V/7-AAD Apoptosis Detection Kit (BD, biosciences) according to the manufacturer’s instructions. Detection and quantification of apoptotic cells were taken by flow cytometry analysis (Beckman Coulter FC500).

### Ethical statement

All experimental procedures were approved by the Ethics Committee of Beijing Anzhen Hospital.

### Statistical analysis

All numeric data were expressed as mean ± SD. All statistical analyses were performed with Students *t*-test for two independent samples or with a *One Way* or *Two Way* ANOVA followed by a Tukey’s *post-hoc* test using SPSS 23 (IBM, USA). Statistics are separately described in the corresponding figure legends as well. Significant differences are depicted in the figures by graphical representation. p < 0.05 was considered as significant.

### Data availability

The datasets used or analysed during the current study are available from the corresponding author on reasonable request.

## Results

### Pulmonary microvascular endothelial cells from irreversible PAH showed apoptosis resistance

**Fig. 1:**
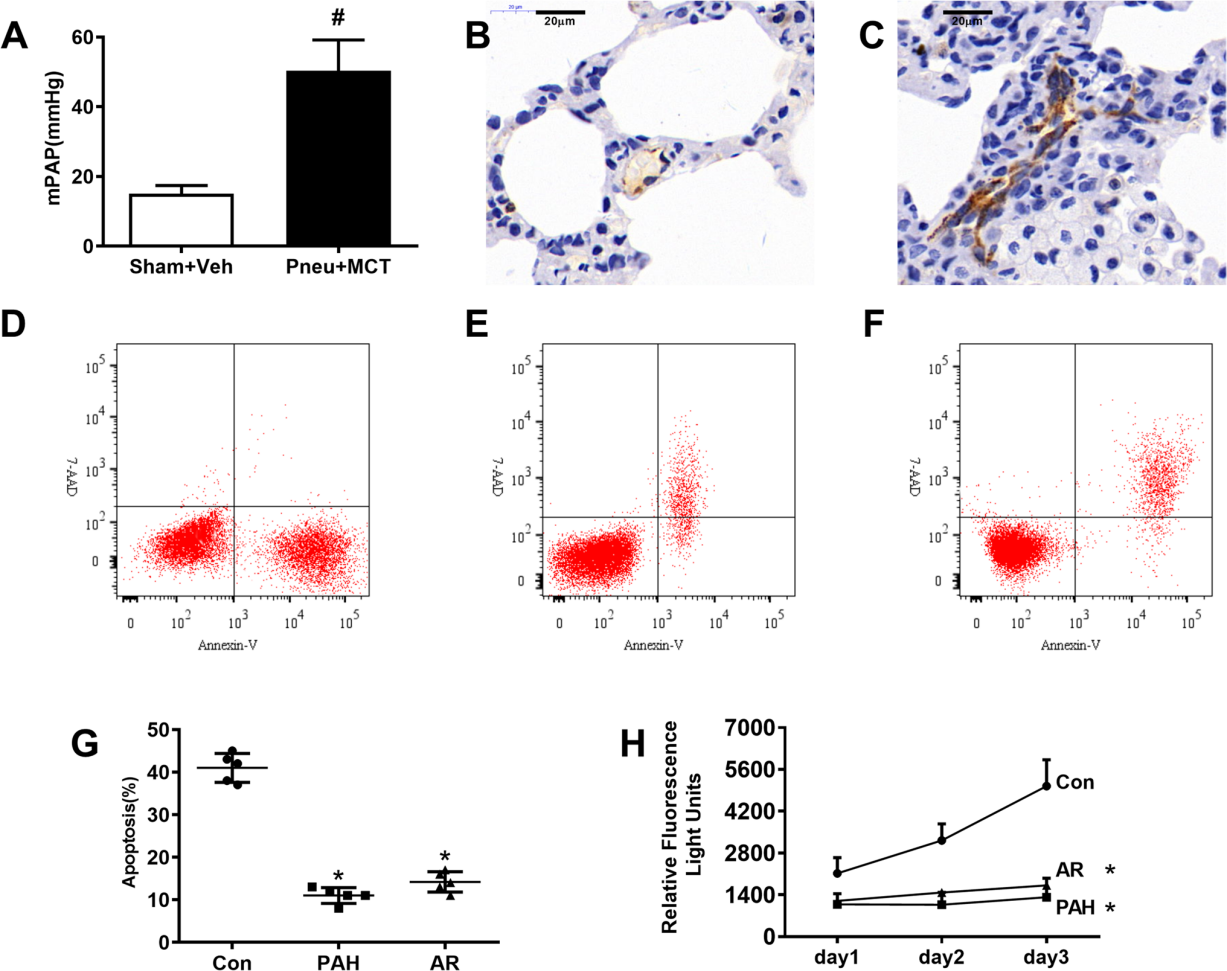
Endothelial cells isolated from pulmonary arteries developing plexiform lesions or treated by hypoxia showed apoptotic resistance. Figure 1A. The mean pulmonary arterial pressure increased significantly higher in Pneu + MCT treated rats than that in Sham + veh treated rats (50.4 ± 8.8 mmHg vs.15 ± 2.4 mmHg, n = 10 in each group, unpaired two-tailed t-test, p < 0.0001) 21 days after treatment. Figure 1B-C, Immunostaining with von Willebrand factor (vWF) showed normal small artery in rats received sham procedure + vehicle treatment (B) and abnormal channels formed by a diffuse population of endothelial cells in rats received Pneu + MCT treatment(C). Figure 1D-G, The PAECs isolated from pulmonary arteries of PAH rats (PAH group) or received hypoxia treatment (AR group) were less apoptotic compared with control rats [Con group (41.00 ± 3.39)%, PAH group(11.00 ± 1.87)%, AR group (14.2 ± 2.39)%, n = 5 in each group, One-way ANOVA Tukey’s multiple comparisons test, PAH vs. Con, p < 0.0001, AR vs. Con, p = 0.0002]. Figure 1H, The caspase-3/caspase-7 activity showed that the reduction in apoptosis occurred over days of culture in PAECs either isolated from PAH rats or caused by hypoxia treatment (n = 5 in each group, Two-way ANOVA Tukey’s multiple comparisons test, relative fluorescence light units: Day 1, PAH vs. Con, p = 0.0192, AR vs. Con, p = 0.0311; Day 2, PAH vs. Con, p = 0.0005, AR vs. Con, p = 0.0024; Day 3, PAH vs. Con, p = 0.0003, AR vs. Con, p = 0.0021). Veh: vehicle, Pneu: pneumectomy, MCT: monocrotaline. #: p < 0.05, vs. sham + veh group, *p < 0.05, vs. con group

Three weeks after monocrotaline injection following left pneumectomy, the rats have developed irreversible PAH, characterizing -with significantly increased mean pulmonary arterial pressure (mPAP) (Fig. 1A). In addition, the immunohistochemistry staining in small pulmonary arteries showed the existence of the vWF-expressing endothelial cells lining vascular channels in PAH rats but not control ones (Fig. 1B, C), indicating that a severe pulmonary proliferative lesion, plexiform-like lesions were formed in the PAH rats. Cultured cells obtained from PAH rats exhibited polygon or fusiform morphologies (Fig Suppl A) and were strongly positive for CD31 and CD 309 as detected by flow cytometry, respectively (Fig Suppl B, C). Decreased cell apoptosis was confirmed by the presence of lower caspase-3/caspase-7 activity among irreversible PAH pulmonary arterial endothelial cells (PAH) cultured for 1 to 3 days compared with that from control rats (Con, Fig. 1H). Furthermore, apoptosis was quantitated by Annexin V-FITC/PI staining based on flow cytometry. Consistently, the rate of apoptosis in PAH group was significantly lower than that of Con group (Fig. 1D, E, G). PACEs treated by hypoxia (5%O_2_) for 24 h induced apoptosis-resistance (AR), with the feature of decreased caspase-3/caspase-7 activity and lower apoptosis rate (Fig. 1F, G, H). The surviving apoptosis resistant cells were harvested and cultured for further use in subsequent experiments.

### Apoptotic-resistance PAECs displayed fragmented mitochondrial morphology

**Fig. 2:**
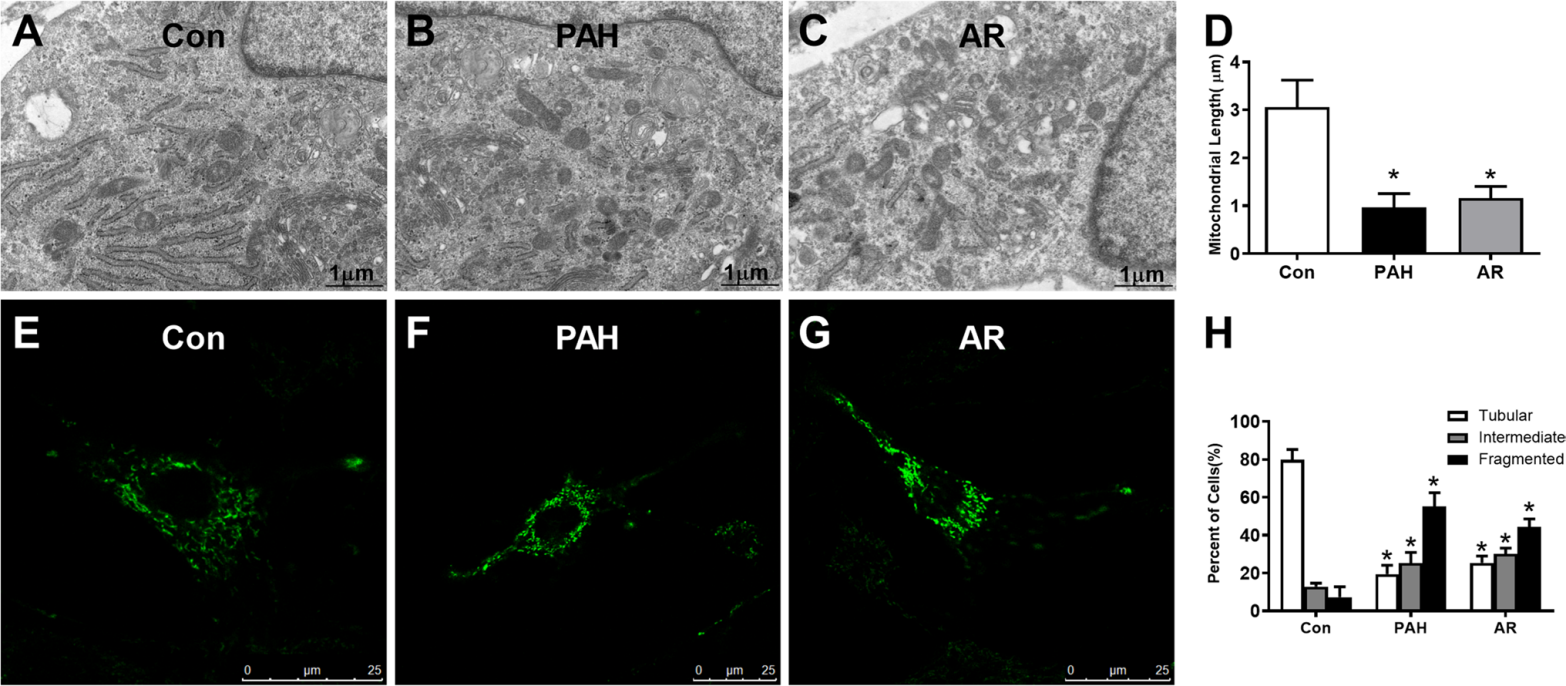
Apoptosis resistant PAECs display fragmented mitochondrial morphology. A-C, Representative images from transmission electron microscopy showed shorter, rounded mitochondria in PAECs from PAH and AR group rats and elongated, tubular structures in PAECs from Con group rats. D, Mitochondria showed significantly shorter in structure in PAECs from both PAH (0.97 ± 0.29 μm, vs. Con group, n = 41 in each group, One-way ANOVA Tukey’s multiple comparisons test, p < 0.0001) and AR group (1.16 ± 0.24 μm, vs. Con group, n = 41 in each group, One-way ANOVA Tukey’s multiple comparisons test, p < 0.0001) rats than that in Con group (3.06 ± 0.56 μm) when analyzed at least 40 mitochondria per experiment. E–G, Representative images of mitochondria with more fragmentation by MitoTracker Green-labeling were presented in PAECs from PAH and AR group, while more tubular mitochondria were displayed in Con group. H, The percentage of cells with fragmented mitochondria in PAECs from PAH [(55.2 ± 7.3)%, vs. Con group, n = 45 in each group, Two-way ANOVA Tukey’s multiple comparisons test, p < 0.0001] and AR group[(44.4 ± 4.2)%, vs. Con group, Two-way ANOVA Tukey’s multiple comparisons test, p < 0.0001] were higher than from Con group [(7.3 ± 5.5)%] when assessed 150 mitochondria in each experimental group. *p < 0.05, vs. con group

As shown in Fig. 2B, the mitochondria in PAH group showed shorter, rounded structures in transmission electron microscopic images, while the ones in control group displayed elongated, tubular structures (Fig. 2B), and the length of mitochondria in PAECs were significantly longer than that in PAH group (Fig. 2D). In addition, the mitochondrial morphology was further analyzed using a mitochondrial marker (Mito Tracker Green) by confocal microscopy. Representative images of the mitochondria are shown in Fig. 2F, and the marked mitochondrial fragmentation was observed in the PAH group compared to the Con group (Fig. 2E). The PAECs in PAH group have a significantly higher percent of fragmented mitochondria than that in Con group (Fig. 2H). In hypoxia induced apoptotic-resistance PAECs (AR group, Fig. 2C, G), mitochondria displayed short isolated dot-like spheres in both transmission electron microscopic and confocal microscopic images, with increased fragmented mitochondria indicating as shorter mitochondria and higher percent of fragmented mitochondria when compared to Con group (Fig. 2D, H).

### Drp1 played a key role in the development of apoptotic-resistance PAECs

**Fig. 3:**
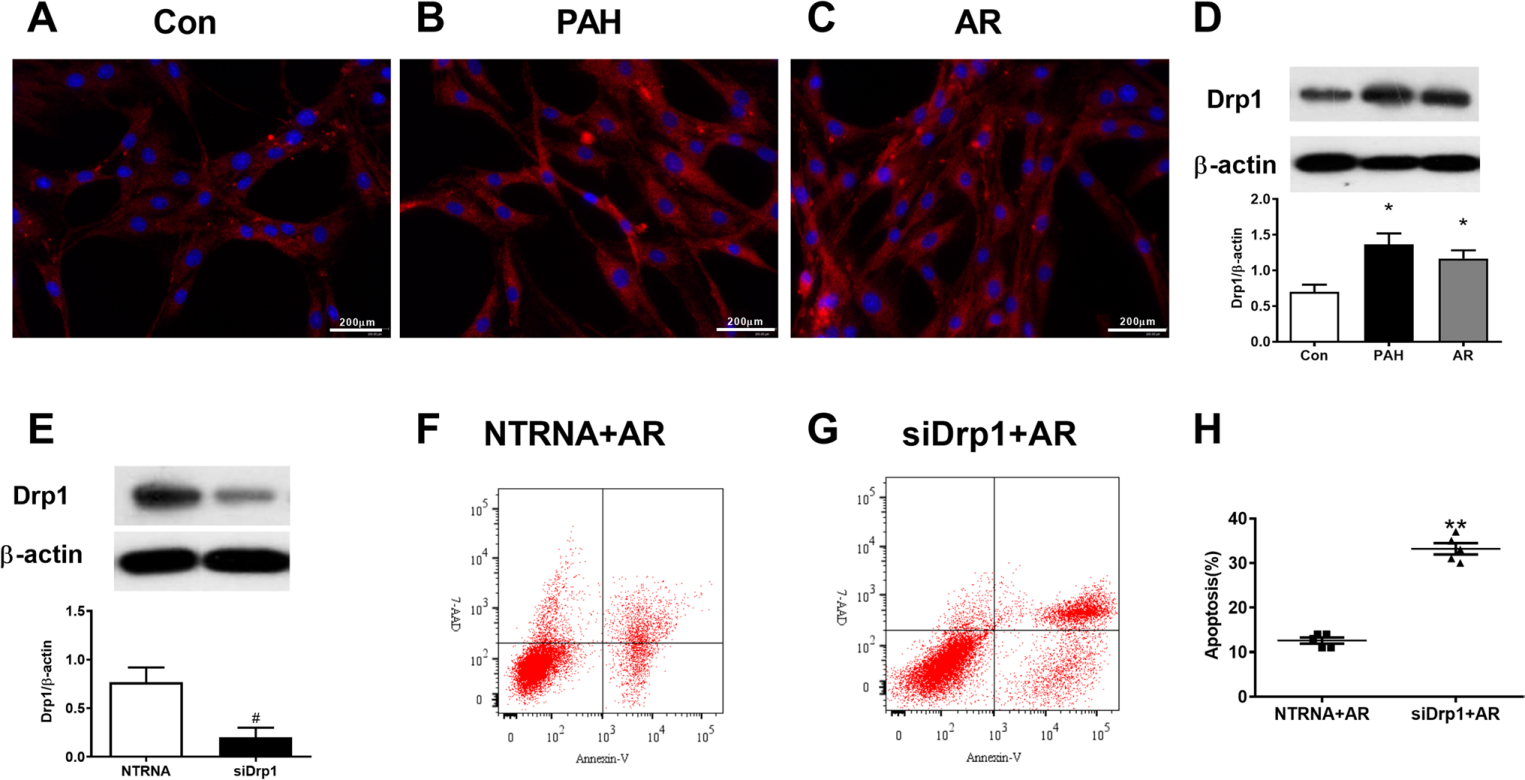
DRP1 was involved in development of apoptosis resistant PAECs. A-C, Immunofluorescence for Drp1 protein expression showed enhanced intensity in PAECs both from PAH and AR group. D, DRP1 protein expression analyzed by western blotting were increased significantly in PAH (vs. Con group, n = 4 in each group, One-way ANOVA Tukey’s multiple comparisons test, p = 0.0115) and AR group (vs. Con group, n = 4 in each group, One-way ANOVA Tukey’s multiple comparisons test, p = 0.0278) when compared to Con group. E, Immunoblot of DRP1 following knockdown via siRNAs compared to nontargeting control siRNA sequence (NTRNA). F–H, The transfection of Drp1-SiRNA blocked the development of apoptosis resistant induced by hypoxia (5% O2) in PAECs as assessed by flow cytometry with PE Annexin V/7-AAD Apoptosis Detection Kit (apoptosis rate: NTRNA + AR, 12.60 ± 1.52 vs. SiDrp1 + AR, 33.20 ± 2.87, n = 5 in each group, unpaired two-tailed t-test, p < 0.0022). *p < 0.05, vs. con group, #p < 0.05, vs. NTRNA group, **p < 0.05, vs. NTRNA + AR group

The role of a key protein Drp1 in regulation of mitochondrial fission was investigated. Immunofluorescence showed that Drp1 was overexpressed in both PAH (Fig. 3B) and AR (Fig. 3C) group. The endogenous protein expression of Drp1 detected by western blotting analysis were significantly higher in both PAH and AR group than that in Con group as well (Fig. 3D).

**Fig. 4:**
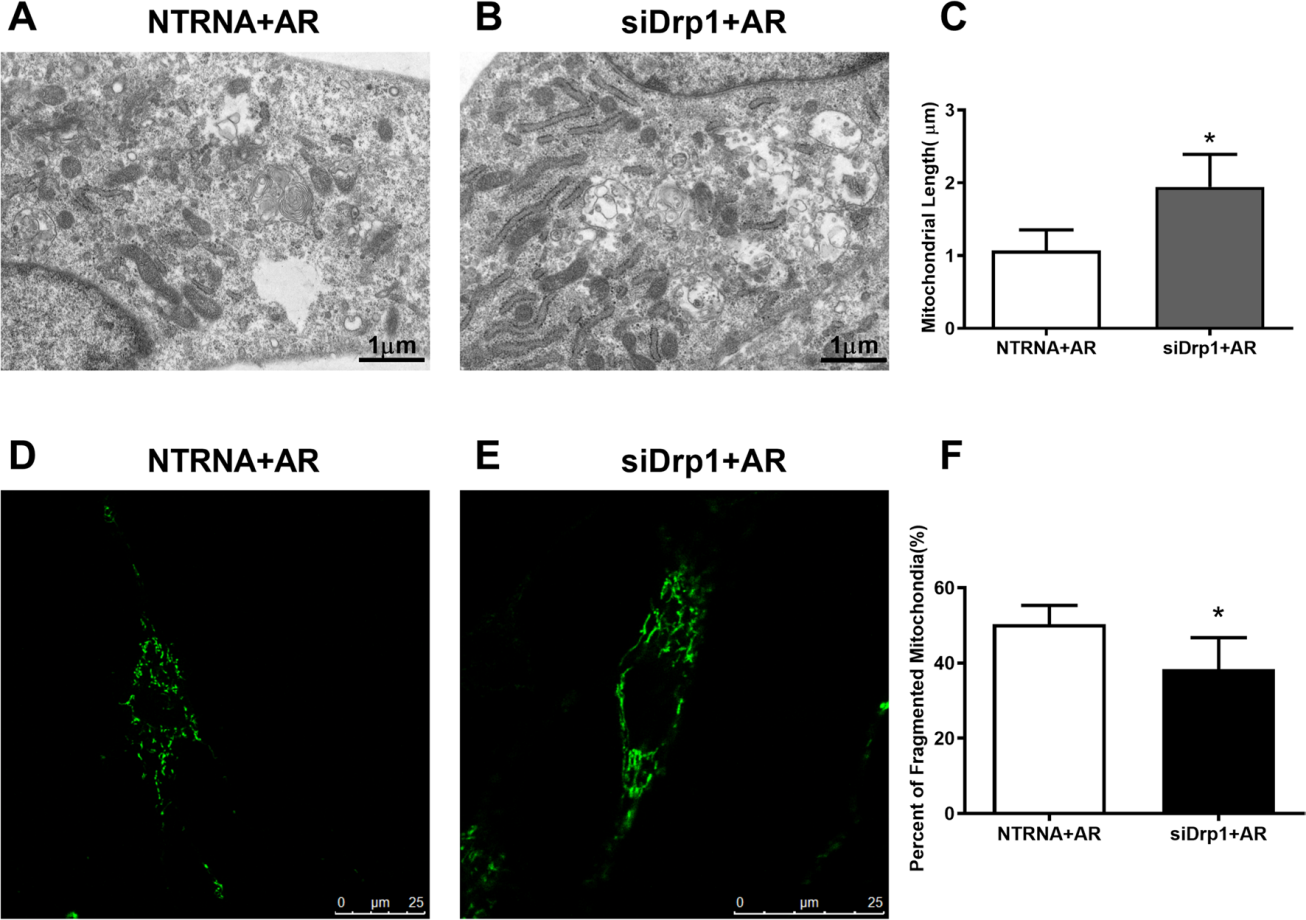
Drp1 knockdown affected the mitochondrial morphology. A-C Transmission electron microscopy images of mitochondria showed that the elongated, tubular mitochondria increased instead of shorter, rounded mitochondria after siDrp1 transfection, indicating as increased length of mitochondria in siDrp1 + AR group than in NTRNA + AR (1.94 ± 0.45 vs. 1.27 ± 0.29, n = 45 in each group, unpaired two-tailed t-test, p < 0.0001). D-F, In hypoxia environment (5% O2), decreased fragmented mitochondria were presented in PAECs with siRNA transfection for Drp1 when compared that received nontargeting control siRNA transfection [Percent of Fragment mitochondria: (50 ± 5)% vs. (38 ± 8.3)%, n = 150 in each group, unpaired two-tailed t-test, p < 0.0001]. *p < 0.05, vs. NTRNA + AR group

**Fig. 5:**
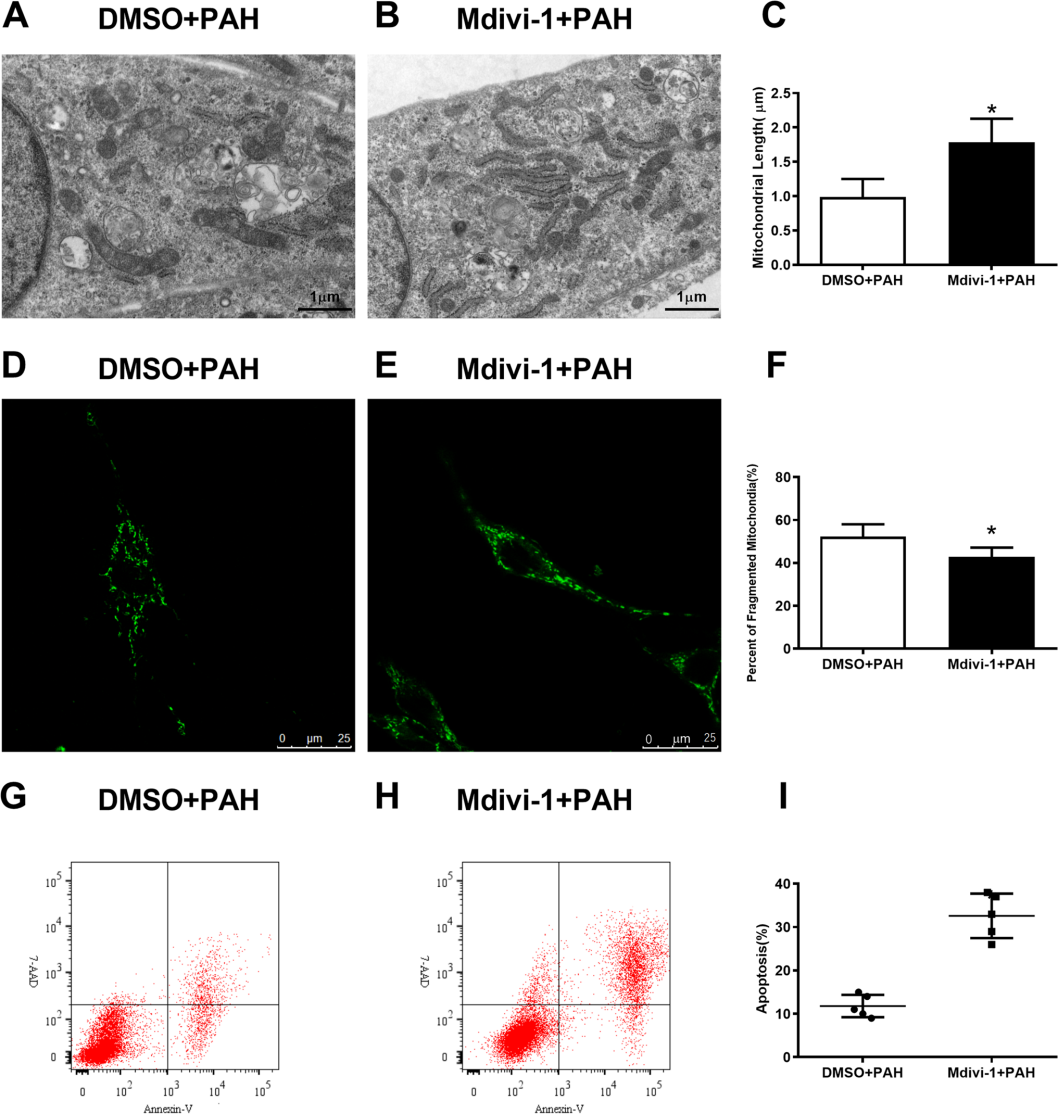
Pharmacologic blockade of DRP1 improved mitochondria fission and apoptosis in PAH PAECs. A-C, Mdivi-1 treatment on PAECs from PAH rats increased the length of mitochondria significantly compared to vehicle treatment as analyzing by Transmission electron microscopy (0.99 ± 0.26 vs. 1.8 ± 0.34, n = 45 in each group, unpaired two-tailed t-test, p < 0.0001). D-F. Reduced mitochondrial fragmentation were displayed in mdivi-1treated-PAH PAECs labeled by MitoTracker Green compared to vehicle treated [percent of fragmentation mitochondria: (52.5 ± 5.86)% vs. (42.8 ± 4.36)%, n = 150 in each group, unpaired two-tailed t-test, p < 0.0001]. G-I, The apoptosis rate were elevated in PAECs from PAH rats after midivi-1 treatment when analyzed using flow cytometry for apoptosis [apoptosis rate: (12.00 ± 2.60)% vs.(33.00 ± 5.10)%, n = 5 in each group, unpaired two-tailed t-test, p = 0.0004].* p < 0.05, vs. DMSO + PAH group

As the up-regulated expression of Drp1 protein in both PAH and AR group, this suggests a potential function for Drp1 in the formation of apoptosis resistant PAECs. Drp1 was silenced in PAECs using SiRNA method, and the expression in PAECs that detected by western blotting was reduced significantly after transfection of Drp1-SiRNA (Fig. 3E). Drp1 silence prevented mitochondrial fission in hypoxia induced apoptosis resistant PAECs which showing as less fragmented and more elongated mitochondria in Drp1 silenced PAECs under hypoxia (5% O_2_) circumstance for 24 h (SiDrp1 + AR, Fig. 4). Consistently, quantitative analysis by flow cytometry indicated that Drp1 silence significantly increased the percentage of apoptosis cells in PAECs after hypoxia treatment (Fig. 3F-H). Similarly, Mdivi-1, a Drp1 inhibitor, rescued the mitochondrial fission and apoptosis resistance in PAECs isolated from PAH rats showing pulmonary plexiform-like lesions, which assessed via the quantification of the mean mitochondrial length (Fig. 5A-C), percent of fragmented mitochondria in cells (Fig. 5D-F) and apoptosis rate (Fig. 5G-I).

### STAT3 activation mediated the expression of Drp1 during the process of apoptotic-resistance PAECs formation

**Fig. 6:**
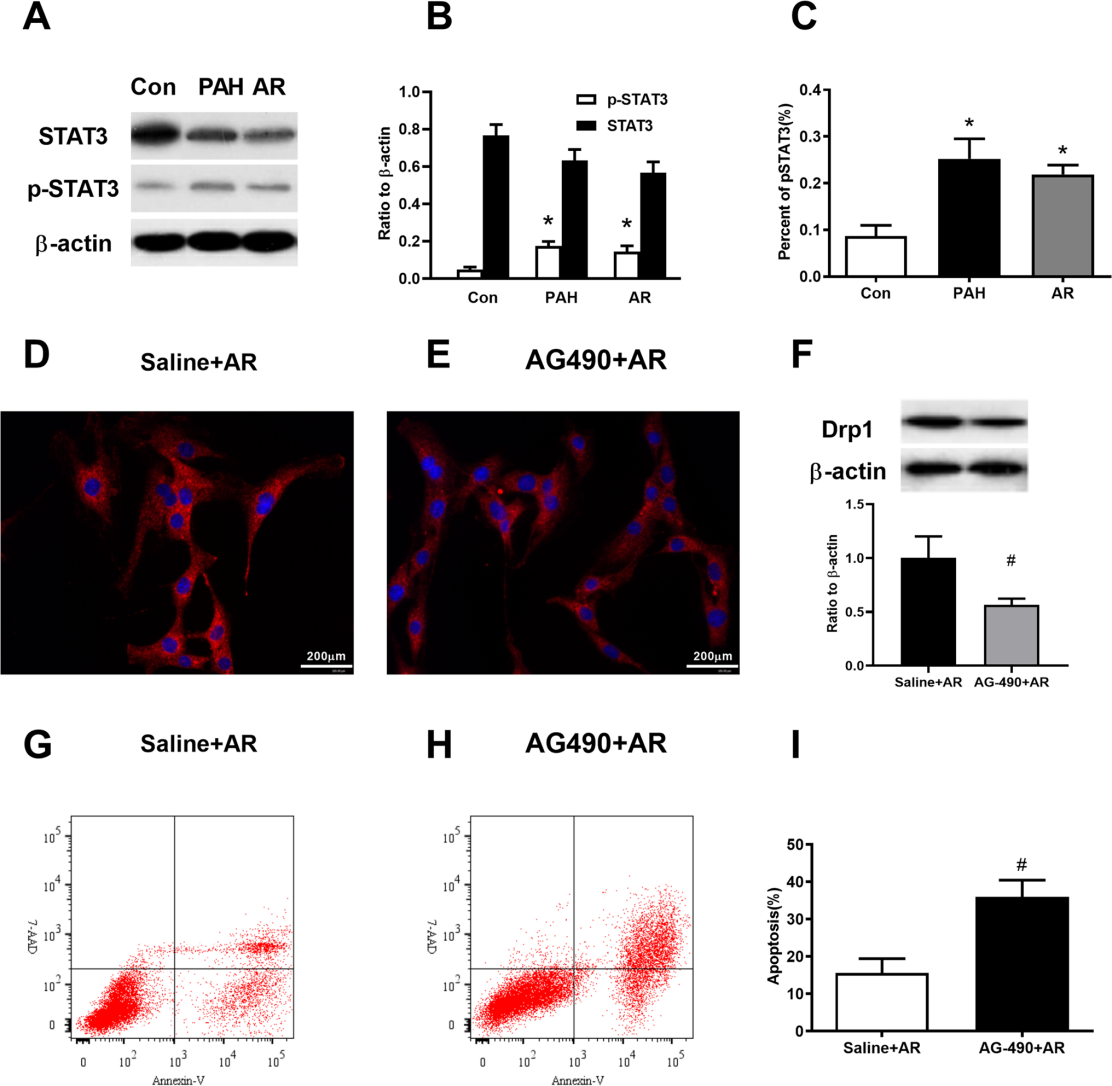
STAT3 phosphorylation at Tyr 705 affected Drp1 protein expression and apoptosis in PAECs under hypoxia. **A**, Immunoblot of STAT3 and STAT3Tyr705 phosphorylation in PAECs from Con, PAH and AR group. **B**. The protein expression of STAT3 among the three groups were similar with statistical difference (n = 3 in each group, One-way ANOVA, p = 0.729), while the levels of STAT3 Tyr705 phosphorylation were higher in both PAH and AR group than that in Con group (n = 3 in each group, One-way ANOVA Tukey’s multiple comparisons test, PAH vs. Con, p = 0.0004, and AR vs. Con, p = 0.0008). **C**. PAECs from both PAH and AR group had increased percent of phosphorylated STAT3 Tyr705 compared with controls (n = 3 in each group, One-way ANOVA Tukey’s multiple comparisons test, PAH vs. Con, p = 0.0014, and AR vs. Con, p = 0.0043). **D**-**E**, Representative immunofluorescence images showed weakened Drp1 protein expression in PAECs with AG-490 (an inhibitor of STAT3Tyr705 phosphorylation) treatment under hypoxia (5% O2). **F**, The decreased Drp1 expression in PAECs received AG-490 treatment under hypoxia compared to receive vehicle treatment under hypoxia (n = 4 in each group, unpaired two-tailed t-test, p = 0.0401). **G**-**I**, The AG-490 treated-PAECs under hypoxia showed increased apoptosis than vehicle-treated PAECs as assessing by flow cytometry [(13.60 ± 3.85)% vs.(36.0 ± 4.47)%, n = 4 in each group, unpaired two-tailed t-test, p = 0.0406]. *p < 0.05, vs. Con group, #p < 0.05, vs. saline + AR group

Phosphorylation of STAT3 plays a major role in preserving mitochondrial function in endothelial cells (Banerjee et al. 2017), here, the effects of phosphorylated STAT3 on mitochondrial fission were investigated in apoptotic-resistance PAECs. The expression of STAT3 in Con group, PAH group and AR group were similar without significant difference among them (Fig.6A-B), while apoptosis resistant PAECs (both PAH group and AR group) have significantly higher phosphorylated STAT3 on Tyr 705 than Con group (Fig. 6A-C). An inhibitor of STAT3 phosphorylation, AG490, prevented the overexpression of Drp1 in PAECs received hypoxia treatment for 24 h (Fig. 6D-F), in addition, the apoptosis in AG-490 treated PAECs under hypoxia environment increased significantly when compared to AR group (Fig. 6G-I).

### Discussion

Recently, mitochondrial dysfunction has become the focus of intensive investigation in PAH, and enhanced mitochondrial fragmentation has also been found in pulmonary arterial smooth muscle cells and right ventricle from animal models of PAH (Culley and Chan 2018). However, there are needsto characterize mitochondria involving in progressive increase in pulmonary vascular resistance due to pathologic remodeling of the pulmonary vasculature, and the effects on endothelial cells remain one of uncharted territory for future discovery (Marshall et al. 2018). We reported that apoptosis-resistant PAECs either from PAH rats developing plexiform-like lesions or induced by hypoxia showed increased mitochondrial fragmentation. The fragmented phenotype in PAECs was associated with up-regulation of the mitochondrial fission regulator Drp1, and Drp1 silence using siRNA or pharmacological blocked by Mdivi-1 prevented the development of mitochondrial fission. Furtherly, activation of STAT3 via phosphorylation at Tyr705 has been proved to play a crucial role in Drp1-mediated mitochondrial fission in the apoptosis-resistant PAECs, providing a basic but novel mechanism underlying the development of PAH.

Irreversible PAH is characterized by plexiform vascular lesions in pulmonary arterioles, which are hypothesized to arise from apoptosis-resistant PAECs (Masri et al. 2007). The young rats received left pneumectomy and monocrotaline injection, following White RJ et al.’s method (White et al. 2007) developed plexiform lesions as identified by immunohistochemical staining with vWF. Primary cultures of PAECs from the animal model showed decreased apoptosis, which confirmed again that apoptosis-resistant PAECs were the pathological hallmark of irreversible PAH. To explore the mechanisms, apoptosis-resistant PAECs were also induced by hypoxia treatment according to Yongmei Cao et al.’s method (Cao et al. 2016).

Although there are several molecules involved in the process of mitochondrial fission, Drp1 is indispensable during mammalian mitochondrial fission. Drp1-mediated mitochondrial fission is intricately related to many distinct pathological conditions, such as cancer (Altieri 2019), neurodegenerative diseases (Alexiou et al. 2019) and cardiovascular diseases. It is believed that Drp1 contributes to the process of apoptosis suppression of pulmonary arterial smooth muscle cells (Zhang et al. 2016) and angiogenesis of PAECs (Shen et al. 2015) and plays multiple roles in pulmonary vascular remodeling. Here we found that impaired mitochondrial dynamics and excessive mitochondrial fission induced by Drp1 overexpression could be an important mechanism in apoptosis-resistant PAECs. First, the percent of fragmented mitochondria increased in PAECs either from irreversible PAH rats or after hypoxia treatment, meanwhile, the length of mitochondria in these two types of apoptosis-resistant PAECs decreased obviously. Second, the increased mitochondrial fission was associated with overexpression of Drp1 by western blotting analysis, and targeting Drp1 using siRNA and pharmacological blocker Midivi-1 inhibited the mitochondrial fission and following apoptosis-resistance.

As a key fission protein in mitochondrial dynamics, Drp1 was involved in pathological injury of endothelial cells in many cardiovascular diseases through influencing cellular energy, ROS generation, intracellular calcium levels, apoptogenic protein production, and so on. In Kawasaki disease murine model, mitochondrial dysregulated fission caused by Drp-1 overexpression precipitated the arterial endothelial cells injury, which could be reversed by Masitinib targeting Drp1(An et al. 2023). Similarly, Drp1 was vital in mediating the overexpression of miR-199b-5p regulated oxidized low-density lipoprotein–induced mitochondrial dysfunction and apoptotic effects in human umbilical vein endothelial cells by targeting AKAP1-mediated mitochondrial fission (Cui et al. 2022). The endothelial cell apoptosis associated with the pathophysiology of atherosclerosis could be alleviated by Mdivi-1, an inhibitor of Drp1, through inhibiting mitochondrial fission. Interestingly, a classical anti-atherosclerotic drug, atorvastatin, prominently alleviated the mitochondrial dynamics disorder and vascular endothelial cell injury both in vitro and in vivo via inhibiting the expression of Drp1 (Liu et al. 2023). The role of Drp1 both in endothelial cell-related pathological process and in pulmonary artery muscle smooth cells hyperproliferation in PAH were reported in previous studies, whereas this work advances the mechanisms of Drp1-mediated mitochondrial fission in the development of apoptotic resistance in PAECs in PAH.

STAT3 plays a critical role in the development of apoptosis abnormality in tumor cell lines, which promotes an apoptosis-resistant environment via the activation of survivin, NFAF and Bcl-2 (Marshall et al. 2018). Increased and sustained STAT3 phosphorylation has been reported in endothelial cells localizing in plexiform lesions of idiopathic human lungs as well as in PAECs from idiopathic PAH human lungs. We reported that apoptosis-resistant PAECs isolated from monocrotaline injection following left pneumonectomy-induced PAH rats or induced by hypoxia treatment demonstrated increased phosphorylation of STAT3 at Tyr 705, and when STAT3 phosphorylation inhibited by AG-490, the apoptosis increased in PAECs cultured in a 5% O2 environment. These findings confirmed the role of Tyr 705 STAT3 phosphorylation in the regulation of apoptosis in a hypoxia-induced apoptosis resistant PAECs.

Recently, several studies have demonstrated that STAT3 is involved in regulating mitochondrial function, either via its transcriptional activity or independent of its transcriptional activity, but the report of its regulation in mitochondrial dynamics is rare. This year, Zhou K et al. firstly showed that JAK2/STAT3 pathway played a key role in regulating mitochondrial dynamics in microglia through regulation of Drp1 (Zhou et al. 2019). Consistently, we found that, in the development of apoptosis resistant PAECs, Tyr 705 STAT3 phosphorylation played a crucial role in mediating mitochondrial fission through affecting Drp1 expression. STAT3 activation was associated with overexpression of Drp1 in both PAECs isolated from PAH rat lung and apoptosis resistant PAECs induced by hypoxia treatment, while AG490 prevented the phosphorylation of STAT3 at tyr705 in hypoxia-induced apoptosis resistant PAECs, and those were associated with the decreased expression of Drp1 protein. Furtherly, Drp1 silencing or blocked by Midivi-1 in hypoxia-treated PAECs showed similar results of apoptosis as AG490 treatment, validating Drp1 regulation by the STAT3 phosphorylation.

### Limitations

Most studies indicated that phosphorylation is the major modification for enhancing the effects of Drp1, but in present study, we showed that the overexpression of Drp1 is one of the mechanisms in the regulation of mitochondrial fission after activation of STAT3 during the development of apoptosis resistance in PAECs, therefore, the phosphorylation or other type of post-translation modifications for Drp1 may need to further investigate. Similar result has been reported by Marsboom et al., in which the upregulation of total Drp1 was involved in driving mitochondrial fission (Marsboom et al. 2012).

## Conclusion

Current therapies do not cure the disease, and the study of the underlying pathogenesis from the pathological features of the constructive cells will advance the therapeutic target for management of PAH. Here we showed that STAT3 activation involved in the Drp1-mediated mitochondrial fission during the development of apoptosis resistant in PAECs which played a central role in the pathogenesis of pulmonary remodeling, and inhibition of STAT3 phosphorylation or Drp1 can induce apoptosis in PAECs under hypoxia environment. Similar results have not been reported in PAECs, and the current study enriches the cellular mechanisms of Drp1-mediating mitochondrial fission in pulmonary remodeling and provides more evidence for the feasibility of targeting STAT3 or Drp1-mediate mitochondrial fission as a therapeutic option for PAH.

### Supplementary Information

Below is the link to the electronic supplementary material.

Supplementary file1 (DOCX 405 KB)
