# Supplementary material for: STAT3 phosphorylation at Tyr705 affects DRP1 (dynamin-related protein 1) controlled-mitochondrial fission during the development of apoptotic-resistance in pulmonary arterial endothelial cells
Source: Genes Genomics. 2024 May 11;46(7):751–62. doi: 10.1007/s13258-024-01522-w (PMC11208226; doi:10.1007/s13258-024-01522-w)
Supplement: Supplementary file 1 — Supplementary file1 (DOCX 405 KB) [file 13258_2024_1522_MOESM1_ESM.docx]

# SUPPLEMENTAL DATA


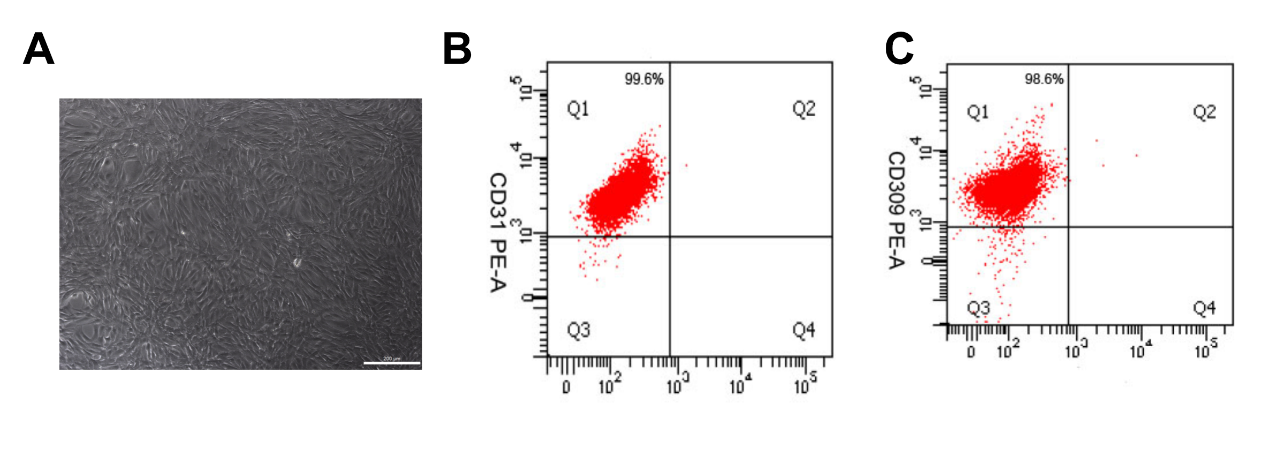


Fig Suppl. The Supplementary Information for the manuscript. A. The primary cultured pulmonary arterial endothelial cells displayed polygon or fusiform morphologies under inverted microscope. B and C. Flow cytometry revealed that 99.6% and 98.6% of pulmonary arterial endothelial cells had positive CD31 and CD34 staining, respectively. D. Full-length bots and details for cropping in the manuscript. Fig 3D (n=4): Con, I(1-4), PAH, II(1-4), AR, III(1-4), β-actin, VIII(1-4); Fig 3E (n=4): NTRNA, VI(5-8), siDrp1VII(5-8); Fig 6A(n=3): STAT3, IV(1-9), p-STAT3, V(1-9), β-actin, VIII(1-9); Fig 6F: saline+AR, II(7-9), AG490+AR, III(7-9), β-actin, VIII(7-9); all western blot analysis were performed simultaneously, and the same band of β-actin (VIII) was used for normalizing, thus the blots of β-actin may overlapped in some figures.
